# Medication history-wide association studies for pharmacovigilance of pregnant patients

**DOI:** 10.1038/s43856-022-00181-w

**Published:** 2022-09-16

**Authors:** Anup P. Challa, Xinnan Niu, Etoi A. Garrison, Sara L. Van Driest, Lisa M. Bastarache, Ethan S. Lippmann, Robert R. Lavieri, Jeffery A. Goldstein, David M. Aronoff

**Affiliations:** 1 Vanderbilt Institute for Clinical and Translational Research, Vanderbilt University Medical Center, Nashville, TN 37203 USA; 2 Department of Chemical and Biomolecular Engineering, Vanderbilt University, Nashville, TN 37212 USA; 3 Department of Biomedical Informatics, Harvard Medical School, Boston, MA 02115 USA; 4 Department of Biomedical Informatics, Vanderbilt University, Nashville, TN 37203 USA; 5 Department of Obstetrics and Gynecology, Vanderbilt University Medical Center, Nashville, TN 37203 USA; 6 Department of Pediatrics, Vanderbilt University Medical Center, Nashville, TN 37232 USA; 7 Department of Medicine, Vanderbilt University Medical Center, Nashville, TN 37203 USA; 8 Department of Pathology, Northwestern University, Chicago, IL 60611 USA; 9 Department of Pathology, Microbiology and Immunology, Vanderbilt University Medical Center, Nashville, TN 37203 USA; 10 Present Address: Department of Medicine, Indiana University School of Medicine, Indianapolis, IN 46202 USA

**Keywords:** Adverse effects, Drug development

## Abstract

**Background:**

Systematic exclusion of pregnant people from interventional clinical trials has created a public health emergency for millions of patients through a dearth of robust safety data for common drugs.

**Methods:**

We harnessed an enterprise collection of 2.8 M electronic health records (EHRs) from routine care, leveraging data linkages between mothers and their babies to detect drug safety signals in this population at full scale. Our mixed-methods signal detection approach stimulates new hypotheses for post-marketing surveillance agnostically of both drugs and diseases—by identifying 1,054 drugs historically prescribed to pregnant patients; developing a quantitative, medication history-wide association study; and integrating a qualitative evidence synthesis platform using expert clinician review for integration of biomedical specificity—to test the effects of maternal exposure to diverse drugs on the incidence of neurodevelopmental defects in their children.

**Results:**

We replicated known teratogenic risks and existing knowledge on drug structure-related teratogenicity; we also highlight 5 common drug classes for which we believe this work warrants updated assessment of their safety.

**Conclusion:**

Here, we present roots of an agile framework to guide enhanced medication regulations, as well as the ontological and analytical limitations that currently restrict the integration of real-world data into drug safety management during pregnancy. This research is not a replacement for inclusion of pregnant people in prospective clinical studies, but it presents a tractable team science approach to evaluating the utility of EHRs for new regulatory review programs—towards improving the delicate equipoise of accuracy and ethics in assessing drug safety in pregnancy.

## Plain language summary

The exclusion of pregnant people during clinical drug development limits our understanding of drug safety in pregnancy. However, given that many approved medications are prescribed during pregnancy, we studied large sets of data from patient electronic medical records, including mother-baby pairs, to develop these drug safety insights. From 2.8 M medical records, we identified 1,054 drugs prescribed to pregnant patients. Combining computerized analysis with expert clinical review, we confirmed signals previously associated with neurodevelopmental defects in children born to drug-exposed mothers. We subsequently identified 5 commonly prescribed drug classes for which we believe this work warrants updated assessment of their safety. Our approach is not a replacement for inclusion of pregnant people in clinical trials but presents a new application of existing medical record data to improve the assessment of drug safety in pregnancy.

## Abstract (web summary)

Challa et al. used 2.8 million electronic health records for proactive pharmacovigilance program development, by rapidly generating and validating hypotheses between medication use during pregnancy and neonatal neurodevelopmental defects. They identify five drug classes for which deeper safety signal evaluation may improve product marketing.

## Introduction

At the point of care, pregnant patients are a complex population: physicians must exercise caution in prescribing many common drugs to these patients, given the risks of toxicity for their developing fetuses^1^. However, consideration of fetal toxicity in drug development is largely irregular. While teratogenicity scores established by regulatory agencies like United States Food and Drug Administration (FDA) are discrete, these criteria provide little concrete distinction among score classes, making it difficult for drug developers to accurately gauge the fetal toxicity risks of a molecule^2^. FDA’s updated teratology assessment guidelines in the 2014 Pregnancy and Lactation Labeling Rule aimed to increase the contextual relevance of developmental toxicity evaluation, but this guidance has been slow to translate to evaluative change at the point of care, which remains largely aligned with the previous five-pronged letter scale^3,4^. The result is a vicious cycle that promotes the approval of drugs without adequate data on their safety and efficacy in pregnant populations, as expectant patients are routinely excluded from clinical trials, out of concern for fetal harm upon exposure to drugs with uncertain, pre-clinical teratogenicity data. In fact, of 213 new drugs approved by FDA between 2003 and 2012, only 5% contained human data in the pregnancy section of their labels^5^. These factors have created a substantial gap in knowledge on pharmacotherapy for diseases during pregnancy, restricting the number of treatments available to this population through insufficient data on the pharmacodynamics and pharmacokinetics (PK) of many maternal medication exposures. At the bedside, the result is undertreatment of chronic and acute illnesses in pregnant people from obstetricians’ cautious fears of causing harm to their patients, alongside the increased risk of harm to fetuses from necessary prescriptions^6^.

While the ethics of excluding pregnant people from randomized, controlled drug trials (RCTs) remain in debate^7–9^, the ongoing unavailability of relevant drug safety and efficacy information underscores an urgent need for new methods to rapidly assess this information, to improve the quality of care for these underserved patients, and to ensure health equity for this complex population through contemporaneous drug labeling and marketing efforts. Such an opportunity for the discovery of drug safety insights for pregnant patients may be available through strategic analysis of large numbers of existing healthcare documents like electronic health records (EHRs) that were collected during routine patient care. Collectively, EHRs can uniquely replicate the natural history of pregnancy by linking medical information of pregnant patients and their neonates, such as mothers’ prescriptions (while expectant) and the perinatal diseases of their children^10–12^. This information allows for the creation of a unique framework of relational knowledge generation. Namely, EHR data may be stratified into distinct cohorts by patients’ documented exposure—or lack thereof—to a drug of interest, facilitating the development of an inferential model to relate incidences of maternal drug exposure and neonatal disease^11^. While these experiments are not a replacement for prospective safety data generation through the inclusion of pregnant people in clinical trials, the above platform of safety signal detection presents an ethical way of studying the effects of drug exposure in pregnant people with human data, on a significant scale and across all drug classes.

Existing literature that describes the safety of most drugs potentially prescriptible in pregnancy remains overwhelmed by conflicting studies—the majority of which only present results from pre-clinical animal models of drug testing and the minority of which are empirical case reports or case series among relatively few patients^13^. Deciding to prescribe a drug to a pregnant patient involves balanced evaluation of the patient’s need for treatment (drug efficacy) and the risk of injury to the patient’s fetus (drug safety). However, providers cannot make these informed decisions without robust and definitive safety data.

Previous work that has attempted to clarify knowledge on drug safety in pregnant patients has relied on observational and retrospective analyses of databases like public insurance claims, measuring the significance in the coincidence of a neonatal disease of interest and prescription of a drug of interest to the neonates’ mothers^10,14^. While these studies have added new—and often valuable—narratives of drug safety to the literature, our research is innovative because it uses EHR data, attempts relational inference, and probes such drug-disease relationships at scale. Collectively, these factors allow us to advance the ontological reliability and epistemological robustness of data-driven studies of adverse pregnancy outcomes^11^.

Our research makes use of a database of 2.8 M EHRs at Vanderbilt University Medical Center (VUMC) to curate our experimental cohorts. The data innovation in studying EHR data over evaluating public insurance claims is that this choice mitigates significant demographic biases (e.g., poverty) that are present within public payor records. Overcoming the effects of such potentially confounding variables requires the integration of advanced methods of propensity scoring (PS) to properly evaluate the coincidence of maternal drug exposure and pediatric disease, which defines the key algorithmic design principle of parsimony and results in poor model performance^15^. In contrast, VUMC is an urban medical center that features a demographically diverse patient population, as previous studies using these EHR data affirm^16^. Indeed, self-reporting patient registries—another popular choice for observational data to study health outcomes in pregnancy—are also inherently limited in their integrity, as patients are often unreliable historians of their own care^17^. In contrast, our study promotes data integrity by studying provider-maintained healthcare information.

Technical innovation in this project also rests within the rigor of the analytical methods we employ^11^. We apply a mode of systematic, relational inference to maternal drug exposure and perinatal disease that we believe is more directly and appropriately aligned with the etiology of drug-associated birth defects, compared to the highly coincidental frameworks that dominate the literature. We achieved inference suggestive of causality through harmonizing the validated phenome-wide association (PheWAS), which was originally developed at VUMC to discover genetic links to clinical phenotypes, with a rigorous, standardized consensus prioritization approach that considered clinical practice and RCT data to move from data-based associations towards etiology discovery^18^. By developing a medication history-wide association study (MedWAS) to suggest pharmacological determinants of neonatal diseases, we optimized on algorithms that underlie PheWAS to explore nascent patterns across the drug-disease hypotheses that our model revealed. In this way, we used MedWAS as a method of safety signal detection and management, approaching the design a target trial^11^. Target trials are an epidemiological method of retrospective data analysis that make use of existing clinical information and high-powered statistical algorithms to create artificial subject profiles from all relevant and available patient data within a cohort of interest. This curation then allows for relational analysis of subjects’ drug histories against a morbidity of interest, facilitating potential simulation of a clinical trial when prospective experiments are not feasible^19–21^. The approach in this manuscript alludes to a target trial by following similar approaches to data curation and stratification, statistical inference, and outcomes prioritization, though unlike the archetypal target trial developed by Hernán and Robbins for claims data and consortial data banks^19^, our distributed workflow relies on a single health system’s mother-baby EHRs, meaning that some aspects of our procedure rely on manual evidence synthesis, rather than harnessing end-to-end automation. Furthermore, our approach operates on known patterns of prescriptive behavior in pregnancy to determine treatment-exposed and non-exposed (i.e., “control”) cohorts in our data, providing a very limited basis to claim RCT-like randomization that is naturally resultant from a poor recapitulation of the many reasons why clinicians decide on specific treatments for their pregnant patients, within structured EHR data. Like the target trial, our research does not seek to replace the RCT. Nonetheless, to our knowledge, there have been very few (and relatively small) attempts at EHR-derived safety signal detection evaluating pregnant patients^22^, allowing us to innovate in exploring the power of this approach at scale^11,23^.

Using MedWAS, we present systematic safety signal detection across all drugs prescribed to pregnant people and all diseases within neonatal EHRs at VUMC: herein lies the conceptual innovation of our approach. Historically, researchers studying the safety of pharmacotherapy in pregnancy with statistical methods have communicated through a “one drug—one disease—one publication” model. While this practice provides bandwidth for deep interrogation of a single drug-disease hypothesis, it further diversifies the pool of existing data that remains conflicting and inconsistent, since the methods in such papers can become overfitted for studying the safety of other drugs that are prescriptible in pregnancy. In contrast, our approach is sufficiently reproducible to analyze maternal prescriptions and neonatal diseases across a large healthcare enterprise. We are unaware of such a drug-agnostic and phenotype-agnostic model in the available literature on drug safety in pregnancy.

We have a record of work in using statistical methods like PheWAS to generate strong hypotheses of efficacy for new drug development^12,24,25^. Here, we apply that expertise to construct MedWAS as an innovatively scalable approach for the surveillance of drug safety in pregnancy. We also present potential avenues for complementarity between MedWAS and our previous attempts to develop a machine learning (ML) approach capable of identifying chemical structures that predispose drugs towards increased teratogenic risk when prescribed during pregnancy^26^.

In this study, we identify 1,054 drugs historically prescribed to pregnant patients and develop a quantitative, medication history-wide association study. We integrate a qualitative evidence synthesis platform using expert clinician review for inclusion of biomedical specificity—to test the effects of maternal exposure to diverse drugs on the incidence of neurodevelopmental defects in their children. Not only do the results replicate known teratogenic risks and existing knowledge on drug structure-related teratogenicity; they also highlight 5 common drug classes for which we believe this work warrants updated assessment of their safety. This research is not a replacement for the inclusion of pregnant people in prospective clinical studies, but presents a tractable team science approach to evaluating the utility of EHRs for new regulatory review programs—towards improving the delicate equipoise of accuracy and ethics in assessing drug safety in pregnancy.

## Methods

The approach that we describe below is an explanatory summary of the data preprocessing (for cohort selection) and informatics procedures (for drug-disease testing) that we provide in cookbook format in the “Supplementary Information” accompanying this manuscript, supporting Supplementary Tables 1–5 in the component “Supplementary Methods” section. A diversity and inclusion report for the maternal and neonatal EHRs we analyzed is also included as Supplementary Table 6 in the “Supplementary Discussion” section.

We tested the hypothesis that MedWAS can effectively establish relational inference between mothers’ exposures to drugs with uncertain safety and perinatal diseases in their neonates. In establishing the feasibility of our tool to accomplish post-market drug surveillance, we restricted ourselves to the analysis of only neurological morbidities as a base case, given that the ontologies that codify these diseases have strong bases of relational logic^27^. We expect the general framework of the analytical and signal evaluation procedures we present here will be analogously applicable to the interrogation of neonatal diseases in other organ systems.

### Ethical review

The Institutional Review Board (IRB) of Vanderbilt University approved the research and deemed that it was exempt from ethical approval and informed consent since it was not deemed to involve human subjects (IRB #191553), given its retrospective, observational nature and use of data collected during routine patient care.

### Cohort selection

To mimic the enrollment of pregnant patients in a drug safety experiment, we used ML to curate and block appropriate treatment and control (drug-exposed vs. not drug-exposed) cohorts across all 1,054 agents that are documented as prescriptions to pregnant patients in eStar, VUMC’s EHR system. A listing of these agents is available as described in Supplementary Methods, supporting Supplementary Data 1. To select our cohorts, we probed VUMC’s Research Derivative (RD), a database of fully identified clinical and administrative information from 2.8 M patients that contains data like International Classification of Disease-9/10 (ICD-9/10) billing codes (which codify nearly all existing human morbidities), patient demographics, lab results, medications, and clinical narratives from five different relational health information systems that source directly from patient care^28,29^. To effectively create experimental cohorts across the agents we probed from these data, we first established the following phenotyping rule as inclusion criteria for patient “enrollment” in treatment and control groups:

Population: RD; Include: Mom/baby link (1 or more), where specified medication (1 or more where date during mother EHR pregnancy=yes) and clinic note in baby EHR suggests record of care (1 or more postpartum).

Herein, our criteria for allocating pregnant patients to a drug treatment group required baseline, confirmed pregnancy among all candidate mothers, with a record of at least one prescription of the specified drug in the mother’s EHR during their entire gestational period and live-born delivery of a neonate who received their own EHR at VUMC (so their health outcomes were available for our analysis). Defining pregnancy and gestational period in a systematic way from the EHR remains a non-standardized analytical practice and therefore required us to develop an inferential approach reliant on a data dictionary of relevant ICD-10 codes for gestational period. For interested readers, we describe this approach in Supplementary Methods, across Supplementary Tables 1–5. We designed our inclusion criteria to maximize the data available to our model, so we could achieve the highest power for demonstrating preliminary proof of concept for our approach. Herein, we harnessed downstream evidence synthesis to vet our outcome associations, rather than establishing very tight inclusion (and exclusion) criteria a priori to mitigate confounders.

### Data curation

Next, we leveraged a suite of natural language processing (NLP) tools to extract phenotypic attributes and maternal drug exposures from narrative EHR data among all patients within the 94,872 EHRs (48,434 mother-baby EHR pairs) who met our inclusion criteria for at least one study drug. These tools included a general-purpose NLP tool (the 2015-indexed version of KnowledgeMap concept identifier (KMCI)^30,31^, available through https://www.vumc.org/cpm/cpm-blog/kmci-knowledgemap-concept-indexer), ML-based clinical-note section tagger (the 2010-indexed version of SecTag^32,33^, available to download at https://www.vumc.org/cpm/cpm-blog/sectag-tagging-clinical-note-section-headers), and version 1.3 of MedEx, an NLP algorithm for identifying medication exposures within free clinical text^32,34^ (available to download at https://sbmi.uth.edu/ccb/resources/medex.htm). KMCI identifies Unified Medical Language System concepts^35^ using a shallow parser, word sense disambiguation, and semantic regularization, and includes a module to identify negation^30^. MedEx uses context-free grammar and a rule-based approach to extract detailed medication information (including dose, frequency, and route) from free text. MedEx encodes an ingredient barcode for all drugs, such that drug mentions extracted from EHRs are continuously linked to existing drug ontologies from which additional pharmacological data may be mined (e.g., RxNorm concept unique identifier^36^)^32,34^. These standardized systems have been used to process more than 60 million documents at Vanderbilt and elsewhere. Here, we used them to capture all drug mentions and available ICD-9/10 codes and to facilitate requisite matching of free-text disease terms to concept unique identifiers for candidate mothers and their linked neonates, as well as to extract all available demographic information for “enrolled” mothers and babies. Enacted across all combinations of diseases and maternal drug histories in our population, our workflow enabled the curation and stratification of patient data to empower >1.7 M combinatorial drug-disease association experiments, as we describe below.

### Implementation of MedWAS

PheWAS is a common, systematic ML approach to unearth associations between disease and genetic variants and to discover pleiotropy using EHR data linked to DNA. It is a method that scans phenomic data for genetic associations using Phecodes mapped to ICD-9/10 codes from the EHR. Multiple publications demonstrate that PheWAS is a feasible method to rapidly generate hypotheses on the underpinnings of disease^18,37–40^. We repurposed the PheWAS framework to develop an innovative MedWAS, in identifying the extent to which the perinatal phenotypes in our cohorts are plausibly related to exposure to the drugs in each simulated safety experiment’s treatment group. Herein, our proof-of-concept MedWAS model took an input of babies’ neurological diseases from all mother-baby cohorts we constructed and outputted the maternal medication exposures putatively related to babies’ phenotypes. While it is easiest to envision our platform through the canonical stratification of mother-baby cohorts by maternal drug exposure, our adoption of neonatal disease-contingent inference across treatment-defined maternal cohorts allowed us to develop capacity for discovery of multiple drug exposures as etiologies for our phenotypes of interest.

MedWAS operated in direct analogy to PheWAS by using its component logistic classification methods (logit) to identify neonatal disease as a function of maternal exposure to a drug of interest and by reporting a *p*-value for each of these drug-disease tests that reflected the strength of logit alignment after correction for multiple testing of a drug across all neonatal diseases in our cohorts. In doing this across 1,054 native maternal drug exposures and the neurological subset of 1,678 EHR-embedded phenotypes—first, on a pilot-scale, with 5.7 K EHR pairs, and subsequently on our full data set of 49 K mother-baby dyads—each experiment was controlled by cases of neonatal disease linked to pregnant patients without a record of exposure to the test drug. Herein, we also computed an odds ratio (OR) as a proxy for the effect size of hypothetical drug-disease enrichment across each of our tested case and control populations. Because there are known associations among the representations of input and output data and PheWAS model performance^38–40^, we iteratively assessed MedWAS performance with several standard representations of the drug and disease data (i.e., different levels of Anatomical Therapeutic Chemical (ATC) codes for drug entities^41^ and Phecodes and ICD-9/10 codes for diseases^42^) from our cohorts to prevent confounding of our results by data type. The list of 1,678 Phecodes we employed is publicly accessible through the open-source code for version 0.12.3 of the PheWAS package (see https://github.com/PheWAS/PheWAS).

### Hypothesis Prioritization

While the explicit goal of our work was to establish a platform for generating hypotheses of drug safety that may be pursued in more targeted studies in the future, we affirm that a non-deterministic challenge in pursuing our experiments was accurate prioritization of MedWAS’s predicted drug-disease relationships by their clinical, biological, and statistical plausibility, given the number of association tests we executed rapidly within our analytical framework. We attempted to meet this challenge by ranking our results with the following heuristics: concordance with known fetal safety risks from published drug labels, a soft constraint of Bonferroni significance (with correction from baseline *p* ≤ 0.05) and OR > 1, compelling clinical reviews from obstetrician and pediatrician consults on the plausibility of substantially implicated drug prescriptions and teratogenic outcomes, reproducibility between MedWAS outputs and the results from our previous work that identified drug structures linked to adverse birth outcomes^26^, and evidence against “confounding by indication” from harmonizing systematic chart review of mothers’ baseline disease states with knowledge of known vertical disease transmission risks within our treatment cohorts. Our application of the *p*-value as a soft prioritization constraint that complemented systematic review from our clinical stakeholders aligns with guidance to this effect from American Statistical Association^43^.

To parse MedWAS results we believed were not clinically plausible or were potentially confounded, we began by restricting all signals associated to nutraceutical products, as we recognized that patient history-informed capture of food and nutritional supplement use data in the EHR is highly unreliable. These agents are available over-the-counter (OTC) and often incompletely reported by patients, such that mention of the agent does not always imply true exposure during gestation^44^.

### Pediatrics evidence synthesis

Then, we consulted a pediatrician with expertise in clinical pharmacology on our study team to identify neurological Phecodes with unlikely manifestation in the perinatal period; these diseases were mainly neurocognitive (e.g., dyslexia) and therefore excluded from consideration as true model results. Our pediatrics consult further stratified higher-level versions of the phenotype embeddings in our model outcomes as incident in infants, toddlers, school-age children, or adolescents, based on disease pattern presentations from clinical practice. Consequently, we excluded all outcomes not plausibly detectable in infants.

### Obstetrics evidence synthesis

Following our pediatrician’s review, we consulted a practicing obstetrician on our study team, who has training in clinical pharmacology and maternal-fetal medicine, to identify the plausibility of prescription of the drugs implicated in our model during pregnancy. In completing this review, our obstetrics consultant synthesized knowledge from her own prescriptive practice, prescriptive guidelines from American College of Obstetricians and Gynecologists, Society for Maternal-Fetal Medicine, departmental practice guidelines at Vanderbilt, and clinical decision software (CDS) like UpToDate^4^ and Reprotox^45^ to stratify our signals as “high-yield” and “low-yield” outcomes. We defined high-yield outcomes as those which demonstrated statistical significance, at least 1% coincidence rate between drug prescription and pediatric disease (such that, with our sample sizes of mothers prescribed each drug and neonates born with each disease, we prioritized only non-unary outcomes), and unclear prescriptive recommendations and/or practice guidelines for implicated drugs (e.g., FDA score C and conflicting case reports described in CDS). These drugs also had plausible prescription during the first trimester of pregnancy, when most neurological organ development occurs. Low-yield outcomes included signals rooted in drugs available OTC, such that EHR data on drug use were not reliable for our first-pass analysis, and signals with drugs sparsely prescribed to pregnant patients in the United States of America due to lack of regional drug supply and/or existing guidance against prescription of these drugs during pregnancy. Our consideration of the latter revealed to us that our low-yield signals may be artifactual noise from our inferential approach to defining gestational period, if these drugs appeared in pregnant patients’ EHRs before discontinuation, when providers first learned of their patients’ pregnancies.

Our designation of the yields of our signals was powered by a spreadsheet model we developed, which codified the considerations above by fields including the following: (1) “drug’s original indication” (to help identify potential cases of confounding by maternal morbidity—by which a neonate could inherit the mother’s disease or the drug’s associated adverse outcomes could be sequalae of pre-term birth precipitated by the disease for which the mother is treated); (2) “FDA drug class”; (3) “trimester of prescription”; (4) “intrapartum or immediate postpartum prescription?” (a response of “yes” to this question resulted in a signal’s relative de-prioritization, given our interest in antepartum exposures and the difficulty of perfectly ascertaining gestational period within the EHR); (5) “duration of prescription”.

In an *ad hoc* fashion, both consultants, as well as a pharmacologist, removed drugs from consideration which presented with implausible PK for their associated toxicities (e.g., non-systematic absorption).

**Fig. 1:**
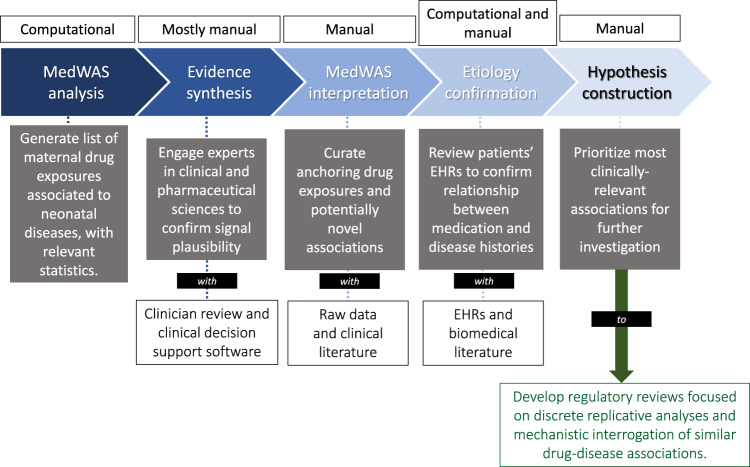
Summary of the process to develop and assure the quality of MedWAS signals—the goal of our approach is to unearth and prioritize drug safety risks, towards generation of the highest quality hypotheses to inform new regulatory review programs. Engagement of obstetric, pharmacological, and regulatory stakeholders is inherent to this process.

Figure 1 provides a summary of our process for developing and vetting MedWAS data.

### Reporting summary

Further information on research design is available in the Nature Research Reporting Summary linked to this article.

## Results

We present MedWAS as a customizable process of generating hypotheses for post-market drug surveillance of drug safety in pregnancy, which takes strategic advantage of the milieu of primary medical care for pregnant patients and the data routinely generated through these encounters. We present key results from our platform below, along with a discussion of the advantages, several limitations, and positive reception of our attempt, which we believe collectively define opportunities for expansion of our approach as a systematic attempt at drug safety assurance that is powered by real-world evidence (RWE).

### Proof of concept

*Prima facie*, we consider MedWAS successful for its robust capacity to accommodate the largescale testing that we envisioned: following our experimental design, pilot testing, and localized sensitivity analyses, we executed 1,770,290 drug-disease experiments using a high-performance cluster with 2,400 processor cores hosted by the Southern Crossroads server^46^ for supercomputing.

As we describe in “Methods,” facing an abundance of generated data, we restricted analysis of the reliability of our results to a single physiological system, to allow for deep contextual analysis. Accordingly, we selected to analyze 1,414 neuroteratogenic signals meeting our aforementioned definition of statistical significance, given expertise in neuropathogenesis within our study team and the spatially and temporally focal nature of many neurodevelopmental anomalies to neurulation^47^, which occurs in the first trimester of pregnancy^48^. In analyzing this functional area, we assume that our insights are sufficiently generalizable to similar physiology in other organ systems, but we also acknowledge that signals among systemic developmental phenotypes may require more formal network analyses. In considering the validation procedures we describe in “Methods” and the evidence requirements we discuss below for signal confirmation, we found that MedWAS performed best on the bases of ATC-4 and Phecode representations of our drug and disease data, respectively. Choosing these representations allowed us to balance data granularity and utility in optimizing model performance, as we tested associations of agent names (but not formulations, as would be available from ATC-5 embeddings) against high-level phenotype codes with logical mappings to the ICD ontology. While drug formulation could present interesting relationships to toxicity (e.g., through elevated concentrations at sensitive physiological sites like the cervix), we consider that our inability to capture this information does not detract from the power of our model to robustly capture associations between maternal drug exposures and adverse neonatal outcomes, as the explicit goal of our model was to discover relationships between the agents mothers consume and adverse outcomes in their neonates. In this way, we consider formulation to have trace effects on fetal toxicity, further given that most agents within our list of agents are consumed orally.

We observed replication of 8 well-known teratogens [phenytoin^49^, valproate^50^, fenofibrate^51^, quinapril^52^, retinoids (tazarotene, vitamin A, and adapalene)^53^, and, topiramate^54^] and 2 teratogens confirmed by our clinical consults [salicylates (phenyl salicylate and salicylic acid)^55,56^] within our MedWAS results. We consider the according 22 signals (sample signal presented within Table 1, below) as positive population controls for our model: when we identified maternal medication history across our health system, we anticipated that such “anchoring” drugs would present with associations to neuroteratogenic outcomes. Negative population controls (i.e., prescription drugs with known protective effects against teratogenicity and/or zero baseline risk of teratogenic outcomes) are inherently uncommon and were therefore difficult for us to develop, further given that protective agents like folate are often taken by all expectant mothers receiving medical care during pregnancy, in addition to other potentially toxic drugs^57^. Herein, our replication of positive control signals through MedWAS allowed us sufficient confidence to procced with analysis of our model outcomes; our intention to develop structured statistical models with inherent controlling—both for each drug-disease test and across our population—also affirms our non-exploratory study design.

**Table 1:** Example MedWAS Outcome^a^: An example MedWAS outcome for a known teratogenic relationship (fetal phenytoin intoxication and chorea) shows statistical significance across several mother-baby pairs, as we expected.

Drug	Disease	*p*	OR	# Disease +	% Disease + with Drug Exposure
Phenytoin	Abnormal involuntary movements	1 × 10^−6^	1.03	195	1

^a^Per the “Data Availability Statement,” row-level positive control data may be available upon request, and the accompanying Supplementary Data 1 contains exposure counts for all drugs we studied.

A collection of 21 similar results across 10 known teratogens supports our claim of proof of concept for our approach^*^.

We considered Bonferroni significance a soft constraint, given increasing consensus that purely statistical significance does not directly imply biological significance—especially in the context of holistic approaches like PheWAS^58^. Instead, we maintained signals with significant *p*-values at a baseline of 95% confidence even if they did not demonstrate Bonferroni significance, relying on the other evidentiary filters we describe below to determine their relative importance. This approach to determining signal significance holds in all places in which we discuss “significant” outcomes within this manuscript.

### Top-Ranking Signals

With a list of convincing drug-disease hypotheses, anchored in statistical significance, literature evidence of preclinical and clinical toxicity, the norms of pediatric and obstetric practice, and replicative case series, we identified several classes of drugs with convincing signals of fetal toxicity that we believe warrant further assessment through more structured epidemiological investigations. These demonstration signals demonstrate the utility of our MedWAS approach to generate a pliable, hypothesis-generating pipeline for the stimulation of post-market regulatory review programs for drug safety in pregnancy.

The following classes of drugs appeared most significantly linked to clusters of adverse neurological Phecodes diagnosable in the perinatal period, including “spina bifida” (*n* = 219 children), “neural tube defects” (*n* = 242 children), epilepsy and convulsions (*n* = 2343 children), abnormal (involuntary) movements (*n* = 602 children), (obstructive) sleep apnea (*n* = 1,376 children), and “infantile cerebral palsy” (*n* = 149 children). We present these relationships not solely from statistical results, but from considering the holistic evidence review that we describe above: (1) With limiting *p* = 4 × 10^−10^ and OR = 1.03, anti-epileptic drugs (including gabapentin, a drug routinely used off-label^59^, and known toxicants like valproate and topiramate^60^, as described above); (2) with limiting *p* = 2 × 10^−7^ and OR = 1.06, psychotropic agents (including alprazolam and other anxiolytic agents, which are often consumed by pregnant patients but have conflicting safety data on their labels^61^); (3) with limiting *p* = 1 × 10^−4^ and OR = 1.02, anti-emetic drugs (including ondansetron, which, while numerously studied in relationship to fetal cardiovascular outcomes^62^, is often consumed in the first trimester and features controversial associations to pediatric central nervous system abnormalities^63,64^); (4) with limiting *p* = 8 × 10^−8^ and OR = 1.50, narcotic analgesics^65^ (including fentanyl, which featured >60% coincidence rate between maternal drug exposure and detrimental neonatal phenotype and occurred with similar disease links and coincidence rates to the opiate antagonist naloxone); (5) with limiting *p* = 4 × 10^−3^ and OR = 1.83, anti-cancer drugs (including tamoxifen, a drug with few uses among pregnant people who choose not to terminate their pregnancies upon a cancer diagnosis—despite its narrow therapeutic index, the drug does not feature a contraindication for pregnancy on its label^66^).

Our teratology quantitative structure-activity relationship model that we describe in “Introduction”^26^ concorded with our present analysis of drugs containing fluoroquinolone and azetidinone motifs, providing us with an additional layer of validative evidence review in support of the performance of our process.

## Discussion

Our results demonstrate that systematic assessment of the pharmacological determinants of pregnancy outcomes is possible via RWE synthesis that repurposes information routinely collected from primary care and is sufficiently flexible to accommodate direct input from the clinical stakeholders who provide care to pregnant patients and their newborn children. In this regard, our process presents the importance of complementing quantitative methods with qualitative evidence, as much of the contextual knowledge on obstetric prescriptive practice and pediatric disease assessment remains unavailable in structured databases. This combination of ML and consensus prioritization among human users for accurate outcomes analysis is archetypal of PheWAS and GWAS approaches, as many previous publications affirm^24,38,67^.

Our signals present opportunities for confirmation and further interrogation through mechanistic models of human development, as well as for more rigorous evaluation through regulatory-facing program development^68–70^. This expansion is facilitated by the availability of an ontology of medical record numbers for patients with each drug exposure and each outcome that we tested, facilitating a review of individual EHRs to confirm true incidence of prescription and disease, as well as to understand confounding variables within the natural history of patients’ care that our quality control system did not consider but may otherwise explain disease signals. These chart reviews are important and must be undertaken rigorously (e.g., through a repeated random sampling approach) for each drug class in which there is interest in the deeper study. In this way, continuously integrating knowledge about the clinical implementation of implicated agents and the manifestations of their related diseases will allow for further specification of our hypothesis generation platform in the more probative research that we have planned in the future. Nonetheless, reliance on medication history in the EHR carries the risk of exposure misclassification, as some pregnant people may not consume medications as prescribed or may self-report their medical history incompletely or inaccurately. In this study, we attempted to mitigate the misclassification risk by parsing signals associated to OTC drugs and nutraceuticals, but we note that the above is an ontological limitation of the EHR. Sources of electronic drug exposure data reflecting increased patient interaction (e.g., therapeutic dose monitoring (TDM) systems) may only be available for certain drugs—and only contain data at specific timepoints—substantially increasing the likelihood of bias from data missingness that is resultant from the limited use of TDM in routine clinical practice.

We again affirm that the goal of our research was the development of an enterprise-wide, hypothesis-generating pipeline of drug safety signals, to inform post-regulatory authorization safety studies. This work does not aim to identify malpractice and does not comprise clinical guidance on prescriptive behavior for pregnant patients.

Despite this orientation and the advantages of our approach, our methods have important limitations that can also spark new research questions. Beyond the randomization barriers we describe in “Introduction,” ontological barriers prevented us from executing PS to explicitly balance our cohorts before attempting MedWAS for the drug-disease inference within closely matched sub-groups. We considered alignment of maternal morbidity to the Charlson comorbidity index^71^ and application of the superficial method of PS developed by Choi et al. for PheWAS-empowered drug development studies^72^, to match mothers with similar baseline medical and demographic histories for comparison through MedWAS. While, if successful, this approach could have increased the resiliency of our analyses to confounding from variables extraneous to the prescription of the drug specified for each experiment, we realized that the number of maternal-fetal linkages from a single academic medical center like ours is too low to achieve the maximal level of controlling in situ. While ~100 K EHRs is a moderately-large data set for implementation of the present research—and represents the data captured from a large, productive medical center—this project demonstrated that execution of our methods with automated controls for confounding by maternal disease history and patient demography requires access to larger databases to prevent attrition of all comparable patient records. Though we could not employ PS in situ, as we had originally hoped, we believe that the evidence synthesis workflow we developed—along with the availability of manual patient chart review modules alongside MedWAS—successfully helped us to address the effects of these potentially confounding variables through our signal vetting and prioritization procedure. In future research, we hope to access larger administrative databases of patient records, so we may better integrate PS into our quantitative process. This access could also allow facilitate testing against more discrete representations of neonatal phenotypes than those encoded by Phecodes.

We affirm throughout this manuscript that a central challenge to studying pregnancy and its outcomes with EHRs is defining the period of gestation. Many EHR systems rely on a “pregnancy flag,” encoding, on the backend, a binary representation of pregnancy status^73^. This flag is problematic^74,75^, as we have noticed in our EHR system that it often triggers by elevation in a patient’s body mass index. Therein, reduced precision from the available marker means that inferential approaches to defining the period of pregnancy are necessary to layer other study elements, such as identifying a patient’s medication history during gestation. Arithmetic approaches—such as subtracting 40 weeks from a patient’s delivery date documented on a labor and delivery form to estimate conception date—are possible for first-pass estimation of gestational period, but they rely on low missingness in delivery date information within a candidate EHR data set. Contrastingly, as we describe in “Methods,” we found that an inferential approach to predicting the first date of gestation is a plausible pathway for pregnancy identification, as data missingness in the extraction of delivery date from the provider-facing EHR to institutionally maintained databases for secondary use is surprisingly significant. Our approach is also more accurate than the arithmetic alternative we describe above, as the former relies on multiple validated signals of obstetric care. We consider this approach more parsimonious than one of systematic data imputation followed by arithmetic determination, and we affirm that detailed informatics of pregnancy determination in the EHR lie outside the scope of the present study (here, we sought a minimum viable solution that could facilitate MedWAS). Similarly, we are unaware of a row-level data source on the natural history of pregnancy that does not present such ontological limitations or that does not require statistical approaches to defining gestational time. Our approach (including gestational period definition and MedWAS execution) is sufficiently robust to work across other data sets aligned to the Observational Medical Outcomes Partnership common data model (CDM)^76^, while enabling our analysts to readily reproduce our phenotyping for future experiments at our institution, given the approach’s training on our EHR data. Nonetheless, refinement of our pregnancy identification approach (to improve its accuracy and robustness for more complex test cases) is an area for future development, which would benefit from collaboration with experts on data standards.

The context of our approach is most immediately aligned with discovering teratogenic associations, with more limited applicability to evaluating potential determinants of general patient safety outside those we considered in this work; this argument is based, in part, on our model’s reliance on teratogens as positive data controls. Also, by design, our study evaluates perinatal outcomes, as testing relationships between *in utero* drug exposures and phenotypes at prolonged stages of the pediatric life course remains highly difficult due to the accumulation of potentially confounding etiologies during the natural history of childhood^77^. Gaps between the informatics strategy underlying our model and the clinical context of prescriptive practice during pregnancy could further restrict the utility of this approach—while some drugs significantly implicated by our model may create transient neurological disturbances in infants, prescriptive practice during pregnancy is most likely to consider long-term risk to the fetus against immediate therapeutic benefit to the mother; this balance is difficult to evaluate with the ontological limitations of EHR data. For these reasons—alongside the primary goal of our work to study safety outcomes—this research does not seek to quantitatively discuss the efficacy vs. safety profiles (i.e., therapeutic indices) of maternal drug exposures.

While we do not consider the boundaries of our phenotyping capabilities as a significant limitation of our approach, we believe that quantifying the extent to which drug exposures during pregnancy can create lifelong disabilities is an important question. Addressing this question remains a “grand challenge” in the fields of pharmacoepidemiology and life course research and therefore warrants the creation of data management infrastructure that is more capable of reliably capturing patients’ childhood progressions through a collection of systems more diverse than EHRs^78^. Nonetheless, we affirm that our decision to implement MedWAS across all pediatric outcomes, with downstream filtration of results to only perinatal outcomes, allowed us to accomplish our goal of discovering potentially iatrogenic etiologies for birth defects, while also allowing us to prospectively harness our data for studies of prenatal determinants of adverse health outcomes that manifest later in childhood, if we can access other data types that we could harmonize with our model’s results.

Similarly, we considered drug exposure during the entire gestational period to enable MedWAS, as we could generate an outcome set of signals associated to a diversity of neonatal diseases from one execution of the model. *Post hoc*, as we evaluated our signals for a neurological disease test case, we restricted our signals to those only associated to drugs with evidence of maternal use during the first trimester of pregnancy. The benefit of this staged approach is that for any future studies that interrogate neonatal diseases associated with pathophysiology that manifests during a different gestational period, we may re-visit one, holistic data set generated by our MedWAS and restrict per a different time window of maternal drug exposure, to hypothesize a list of potential pharmaceutical determinants of that outcome. Herein, we may focus on signal interrogation for new use cases, rather than re-constructing the signal generation phase of our work.

Our understanding of the potential applicability of MedWAS towards new drug development is two-fold. First, we consider that drug candidates within the same class as existing drugs (i.e., chemical structure or biological indication) could feature similar safety profiles, as supported by the teratogenicity QSAR model that our group previously published referenced above^26^. Second, we believe that the development of new therapeutic uses (NTUs) by label expansion (i.e., evaluation of the efficacy of an approved drug for a new indication) could benefit from MedWAS results in the consideration of whether to include pregnant people in prospective experiments for safety evaluation, as well as in determining the applicability of an NTU toward diseases within the pregnant population through leveraging existing, post-marketing data about the drug in its original use^70^. Nonetheless, even within drug classes we evaluated, individual drugs’ PK could vary^79^; therefore, our process is not designed to accommodate safety signal detection before phase IV of drug development.

In keeping with the results of most PheWAS studies, we are aware that the signals generated from this platform are potentially controversial^80^ and that despite our attempts to integrate multiple streams of clinical, statistical, biological, and archival evidence with manual EHR review, several of the hypotheses we generated may be explained by non-pharmacological factors. We believe, however, that the strength of our platform is in the identification of priority areas for post-market review of drug use during pregnancy that is anchored in RWE, that is sufficiently robust to accommodate the diversity of maternal drugs and perinatal diseases that naturally manifest in a large health system, and that is sufficiently parsimonious to allow for process replication in other health enterprises. We consider that the limited preconditioning necessary for the execution of our approach makes it pacakageable, and that the qualitative aspects of our study design allow us to engage necessary clinical stakeholders for drug review more closely than further automated approaches might.

Future research may take forward our high-level identification of potentially unsafe drug classes, through more structured, epidemiological probes of exposure and outcome. Similarly, we believe the standardization of our quantitative process makes it sufficiently pliable to implement at other health systems with the same CDM underlying their EHRs, which we hope will further advance our understanding of the robustness of our hypothesis generation approach, when deployed across multiple sites’ data warehouses.

We envision that this work will allow us to partner with regulators of drug products to develop new programs that harmonize real-world data sources, towards detecting and evaluating safety signals for drugs authorized for use among pregnant patients. Furthermore, as part of a bench-to-bedside initiative to generate more accurate signals of drug safety in the regulatory evaluation of drug products potentially prescriptible to pregnant people, we are currently developing organotypic models of the human placenta^81^ and developing brain^82^ that can allow us to validate our most convincing MedWAS signals on a mechanistic basis.

## Supplementary information

Supplementary information

Supplementary Data 1

Description of Additional Supplementary Files

Reporting Summary

## Data Availability

Disclosure of our MedWAS data, though de-identified and aggregated, is subject to approval and oversight by the Office of Contracts Management (OCM) at VUMC, as our source data is derived from protected health information (PHI), and some drug-disease pairs are individually re-identifiable. Therefore, institutional policies prevent us from publicly releasing our data tables and their annotations in the interest of patient data security, but, within the data sharing regulations of our institution, we have attempted to provide meaningful information on the content and formatting of our outputs throughout this manuscript. Linked to our Supplementary Information file, we have also provided a supplementary data attachment (Supplementary Data 1), which provides a listing of agent tokens resultant from the phenotyping we describe above, alongside the number of pregnant people containing each agent within their EHRs (agents with no more than 5 exposures are censored accordingly within the spreadsheet). We are committed to open-source science and to ensuring the reproducibility of the research we present here; therefore, we are happy to discuss data transfer requests with researchers interested in our results. Interested investigators should contact the Corresponding Authors at the addresses accompanying this manuscript, and they are happy to discuss forwarding such requests to OCM (towards a data use agreement) within 30 days of receiving such a collaboration request.

## Code availability

We leveraged a suite of previously published natural language processing (NLP) tools to extract phenotypic attributes and maternal drug exposures from narrative EHR data; versions, accession links, and source paper citations for these resources (which contain further description of their mechanisms) are provided at the points of their first reference within the text of our manuscript. The PheWAS package in R, version 0.12.3, is open-source and available through the GitHub link provided above, as well as further described in the PheWAS source papers cited throughout this manuscript. The custom implementation of this open-source package for MedWAS is governed by the same disclosure policy as our MedWAS data; access to this code may be requested through the mechanism we describe in “Data Availability” (we describe the execution in R version 3.6.3, via cookbook format, within our accompanying Supplementary Methods).
