# Supplementary material for: Medication history-wide association studies for pharmacovigilance of pregnant patients
Source: Commun Med (Lond). 2022 Sep 16;2:115. doi: 10.1038/s43856-022-00181-w (PMC9481638; doi:10.1038/s43856-022-00181-w)
Supplement: Supplementary file 1 — Supplementary information [file 43856_2022_181_MOESM1_ESM.pdf]

Supplementary Information
Medication History-Wide Association Studies for Pharmacovigilance of Pregnant Patients

Authors: Anup P. Challa[1,2,3,*], Xinnan Niu[4], Etoi A. Garrison[5], Sara L. Van Driest[6,7], Lisa M. Bastarache[4], Ethan S. Lippmann[2], Robert R. Lavieri[1], Jeffery A. Goldstein[8], David M. Aronoff[5,7,9,*]

Affiliations:

[1]Vanderbilt Institute for Clinical and Translational Research, Vanderbilt University Medical Center, Nashville, TN 37203, USA
[2]Department of Chemical and Biomolecular Engineering, Vanderbilt University, Nashville, TN 37212, USA
[3]Department of Biomedical Informatics, Harvard Medical School, Boston, MA 02115, USA
[4]Department of Biomedical Informatics, Vanderbilt University, Nashville, TN 37203, USA
[5]Department of Obstetrics and Gynecology, Vanderbilt University Medical Center, Nashville, TN 37203, USA
[6]Department of Pediatrics, Vanderbilt University Medical Center, Nashville, TN 37232, USA
[7]Department of Medicine, Vanderbilt University Medical Center, Nashville, TN 37203, USA
[8]Department of Pathology, Northwestern University, Chicago, IL 60611, USA
[9]Department of Pathology, Microbiology and Immunology, Vanderbilt University Medical Center, Nashville, TN 37203, USA

*Corresponding authors

Contact:

David M. Aronoff, MD, FIDSA, FAAM	Anup P. Challa, MS, EIT
Indiana University School of Medicine,	Vanderbilt University,
Emerson Hall 305,	PMB 350969,
545 Barnhill Drive,	2301 Vanderbilt Place,
Indianapolis, Indiana 46202,	Nashville, Tennessee 37203,
United States of America	United States of America
Email: aronoff@iu.edu	Email: sb108vg@gmail.com
Tel. (desk): +1-317-274-8438	Tel. (mobile): +1-901-361-6583
Tel. (fax): +1-317-274-1437	

Table of Contents

Item *Page*

Supplementary Notes…………………………………………………………………………...3

Supplementary Methods………………………………………………….………...........4

I. Defining Period of Gestation……………………………………………………...4

II. Collecting Mothers' Medication Data during Gestation………………………..……...7

III. Collecting Children's Phenotype Data…………………………………………………9

IV. Running MedWAS with the PheWAS Package………………………………………10

Supplementary Results…………………………………………...………………………12

Supplementary Discussion……………………………………………………………….…13

Supplementary References……………………………………………………...………..14

Supplementary Notes

We provide the following expanded methods and characterization of our mother-baby cohorts with hope that these data enhance the reproducibility of the results that we detail in our manuscript. Though, as we describe in our manuscript, regulations that protect the security of the patient data that underlie our MedWAS prevent disclosure of the associated outcomes, we hope that this supplement conveys the pliability of our approach. For extraneous data or code requests, please contact the Corresponding Authors at the email addresses provided above.

Supplementary Methods

I. Defining Period of Gestation

Consider a data set in the form of a list of mother and baby link/pair with accompanying demographic data for each pair.

Use ICD-10 encoding of weeks of gestation Z3A* to determine mothers' day 1 gestation and period of gestation (the asterisk symbol after the primary code, Z3A, means to include all of secondary codes after Z3A. Detailed information for Z3A and its secondary codes are accessible here: https://www.icd10data.com/ICD10CM/Codes/Z00-Z99/Z30-Z39/Z3A-.

1. For mothers who got ICD-10 codes for gestation weeks large than 8 weeks and less than 42 weeks (From Z3A.08 to Z3A.42):

    a. Search against the list of mother-baby link to identify mothers who got ICD-10 codes from Z3A.08 to Z3A.42.

    b. Get mothers' earliest and latest coding dates for those codes for each pair of mother and baby pair.

    c. Calculate the days between the latest and earliest ICD-10 code dates and if the calculated days is less than 250 days, derive the 1st day of mothers' gestation by subtracting the latest coding date with the days calculated from the weeks of gestation multiplied by 7 days.

    d. Calculate the period of mothers' gestation by using the first day of mothers' gestation derived from the latest ICD code date and babies' birth date.

    e. For the calculated days from step c > 250 days, derive the first day of mothers' gestation by subtracting the earliest coding date with days calculated from the weeks of gestation multiplied by 7 days.

    f. Calculate the period of mothers' gestation by using the first day of mothers' gestation derived from the earliest ICD code date and babies' birth dates.

2. For mothers who got ICD-10, Z3A.01, less than 8 weeks:

    a. Search against the list of mother-baby pairs to identify mothers who got Z3A.01 less than 8 weeks of gestation.

    b. Get mothers' earliest and latest coding dates for Z3A.01.

    c. Calculate the days between earliest and latest coding dates for Z3A.01.

    d. Keep mothers with 0 < the days between earliest and latest coding dates for Z3A.01 < 56 days.

    e. Determine or keep mothers' day 1 gestation by subtracting the latest coding date with 28 days or 4 weeks if the days (period of gestation) calculated from babies' birth dates to mothers' day 1 gestation is between 150 and 295 days.

3. For mothers who got ICD-10 larger than, Z3A.49, 42 weeks:

    a. Search against the list of mother-baby pairs left over from Step 2 to identify mothers who got Z3A.49, large than 42 weeks gestation of pregnancy.

    b. Get mothers' latest coding dates for Z3A.49.

    c. Estimate the first day of gestation by subtracting the latest coding date with 42 weeks multiplied by 7 plus an extra 4 days.

    d. Calculate mothers' period of gestation by using the first day of gestation and babies' date of birth and only keep the calculated period of gestations larger than 295 and less than 320 days.

Combine the data sets from steps 1, 2, and 3, and denote the resulting data as "mother-baby_icd10."

Now, use the Systemized Nomenclature of Medicine (SNOMED)[1] to determine mothers' day 1 gestation and period of gestation. To implement this approach, EHR data should be stored under the Observational Medical Outcomes Partnership (OMOP) common data model[2].

1. Prepare the list of mother-baby pairs whose day 1 and period of gestation were not determined using above ICD-10 code approach.

2. Collect a list of concept_id from table, CONCEPT, by specifying the field, concept_name having the keywords, ’gestation period’ and the key word, ‘weeks’ as the last word in the value of each concept_name. Also, the field vocabulary_id needs to be specified with “SNOMED” to define the key words search is limited to be from SNOMED.

3. Collect mothers’ IDs, SNOMED codes, condition_source_value, condition_start_date in table CONDITION_OCCURRENCE and then merge them with concept IDs from step 2.

4. Group the records based on mothers’ ID and derive the earliest and latest dates for a mother who got those SNOMED codes.

5. Keep those mothers whose days between earliest and latest date of SNOMED is less than 280 days but larger than 0 days.

6. Deduce mothers’ day 1 of gestation by using the latest date of getting SNOMED to subtract the days calculated from the weeks of gestation multiplied by 7. Then, calculate the period of mothers’ gestation by using babies’ birth dates and the derived mothers’ day 1 of gestation.

7. Also, keep those entries with mothers whose days between earliest and latest date of getting mapped SNOMED is large than 280 days.

8. For mothers coming from step 7, deduce mothers’ day 1 gestation by using the date of earliest getting SNOMED to subtract the days calculated from the weeks of gestation multiplied by 7, and then calculate the period of mothers’ gestation by using babies’ birth date and the derived mothers’ day 1 gestation.

9. Pool datasets from steps 6 and 8 and denote it as “mother-babysnomed.”

Now, use ICD-9 and Current Procedural Terminology (CPT) billing codes to determine mothers’ day 1 gestation and period of gestation.

1. Prepare mother-baby dataset which wasn’t determined by the strategies of using ICD-10 and SNOMED.

2. Search above dataset with ICD-9 codes, v72.42, v22.0, and v22.1 to create a cohort, from which mothers are currently getting positive test results (v72.42) for normal pregnancy (v22.0 and v22.1).

3. Collect data from the cohort created in step 2 by keeping only these mothers who also got CPT codes, '76801' and '76802'.

4. Derive mothers' day 1 gestation by using the date of CPT codes minus 98 days.

5. Calculate the period of mothers' gestation using mothers' day1 gestation and babies' birth date and then only keep the period of mothers' gestation is between 260 and 310 days.

6. Name the dataset as "mother-baby_ICD9_CPT".

Finally, pool the 3 datasets, mother-baby_icd10, mother-baby_snomed, and mother-baby_ICD9_CPT to be the final dataset, which is used for rest of MedWAS analysis.

II. Collecting Mothers' Medication Data during Gestation

Consider the defined mother-baby dataset from section I, which is a list of mother and baby pairs with their demographic information, day 1 gestation, and the estimated period of gestation in days. Here, we need to prepare medication data during the estimated period of mothers' gestation and assume that all EHR data for replicative analyses are implemented using OMOP schema.

1. Specify medication data collection is based on the drug ingredient by limiting CONCEPT_CLASS_ID=' Ingredient'.

2. Collect concept_id and concept_name (drug_ingred) using SQL code like that below.

```
SELECT DESCENDANT_CONCEPT_ID AS CONCEPT_ID,CONCEPT_NAME AS DRUG_INGRED FROM
(select * from (
SELECT DISTINCT CONCEPT_ID,CONCEPT_NAME,CONCEPT_ID AS ANCESTOR_CONCEPT_ID,CONCEPT_CODE
FROM (
select * from concept where
upper(domain_id)like upper('drug%')and
UPPER(CONCEPT_CLASS_id)LIKE UPPER('INGREDIENT%'))D_INGREDIENT)P
```

```
JOIN
CONCEPT_ANCESTOR B
using(ancestor_concept_id))PC_J
```

3. Join or merge patients' EHR medication data in table, `DRUG_EXPOSURE`, with drug_ingred (concept_name) using concept_id from step 2.

4. First, collect mothers' medication data during the period of gestation by merging or joining data from step 3 using mothers' IDs from the defined mother-baby dataset developed in section I.

5. Second, define the window for collecting mothers' medication exposure during pregnancy as 60 days before the first day of mothers' gestation until birth dates.

6. Option #1: to collect mothers' medication data during the period of gestation as binary data, just assign "1" as drug exposure. Or, option #2: to collect mothers' gestational medication data as abundance data, cumulatively count medication exposure times.

Prepare a medication exposure data file in the format of binary or abundance, as in the demonstration tables below (Supplementary Tables 1 and 2).

Note: When dealing with drug's name, eliminate any none (Aa-Zz and 0-9) characters and replace 'white space' with '_' to get final drug names. For example, the drug name, "INFLUENZA VIRUS VACCINE, INACTIVATED A-CALIFORNIA-07-2009 X-179A (H1N1) STRAIN", was changed to "INFLUENZA_VIRUS_VACCINE_INACTIVATED_A_CALIFORNIA_07_2009_X_179A_H1N1_STRAIN."

Supplementary Table 1—Binary Form of Medication Data: Example medication data in binary format (to protect patient privacy, mother-baby linkage identifier redacted in this display)

M_B_ID	ZONISAMIDE	ZOLPIDEM	ZIDOVUDINE
*********_*********	0	0	0
*********_*********	1	1	0
*********_*********	1	0	1

Supplementary Table 2—Abundance Form of Medication Data: Example medication data in abundance format (to protect security of patients' data, mother-baby linkage identifier redacted in display)

M_B_ID	ZONISAMIDE	ZOLPIDEM	ZIDOVUDINE
*********_*********	0	0	0
*********_*********	5	8	0
*********_*********	12	0	36

III. Collecting Children's Phenotype Data

Consider the defined mother-baby dataset from section I, which is a list of mother and baby pairs with their demographic information, day 1 gestation, and period of gestation in days. Here, we identify phenotypes for all candidate children and align the phenotypes to Phecodes.

1. The time window to collect babies' phenotypes is defined as from the date of birth specified to when the babies turn 18 years old.

2. Based on the times defined from step 1, babies' ICD-9 and ICD-10 codes are collected and stored in a CSV file (per the example data in Supplementary Table 3, below) using babies' ID from the defined mother-baby dataset.

Supplementary Table 3—Demonstration of Phenotype Count: Demonstration phenotype data (as ICD-9 or ICD-10 codes) for candidate children—"COUNT" specifies number of instances of code in the child's EHR during the period of observation. To protect security of patients' data, mother-baby linkage identifier is redacted in display.

M_B_ID	VOCABULARY_ID	CODE	COUNT
*********_*********	ICD10CM	Z38.00	70
*********_*********	ICD9CM	754.51	11
*********_*********	ICD10CM	P96.0	420

3. The collected ICD-9 and ICD-10 codes above are converted to Phecodes (per the example data in Supplementary Table 4, below) using the PheWAS package download from the following website: https://www.vumc.org/cpm/center-precision-medicine-

blog/phewas-r-package. The details of converting ICD-9 and ICD-10 codes to Phecode are accessible through the PheWAS package.

Supplementary Table 4—Demonstration of Phecode Alignment: Phenotype data aligned as Phecodes following execution of the PheWAS package—Phecodes converted from the ICD ontology are displayed from the second column onwards. "TRUE" means baby was phenotyped as case for a MedWAS test, "FALSE" stands for baby was phenotyped as control for that test, and "NA" denotes that baby was phenotyped as neither case nor control. To protect security of patients' data, mother-baby linkage identifier is redacted in demonstration.

M_B_ID	748	749	749.1
*********_*********	TRUE	TRUE	NA
*********_*********	FALSE	FALSE	NA
*********_*********	TRUE	NA	NA

IV. Running MedWAS with the PheWAS Package

Consider the medication dataset created from section II, which stored in the format of CSV flat file. The phenotype dataset created from section III is stored as an R object following import and execution of version 0.12.3 of the PheWAS R package. Here, we demonstrate an example of running association analysis between the children's phenotypes and mothers' medication data in binary format during the period of their gestation. Version 3.6.3 of R was the most current at the start of the PheWAS analyses described in our manuscript and supplement.

1. Create a mothers' medication R object by loading the medication dataset file to R using the command below.

```
medx_data_bin <- read.csv("final_medx_flat_data_bin.csv",head=T,sep=",")
```

2. Run MedWAS by looping each drug (from drug 1 to `n`, where `n` denotes the number of the drugs available for MedWAS) in `medx_data_bin` using the command below.

```
drug_pheno_as1-n <- phewas(phenotype_data, drug1-n_data)
```

3. Assign phenotype information for each drug-phenotype association output from step 2 by using the command below.

```
drug_pheno_as1-n <- addPhecodeInfo(drug_pheno_as1-n)
```

4. Output each drug-phenotype association output as CSV files using command below.

```
write.csv (drug_pheno_as1-n, "./output_directory/drug_pheno_as1-n,".csv",sep='')
```

5. Loop each outputted `drug_pheno_as1-n.csv` to remove any row with the eighth field having the value "A," and only keep the fields {2,3,5,6,7,8,9,10,11,12,16}. Finally, merge all fields one file, which is the final MedWAS outcome/data file (see example data in Supplementary Table 5, below)

Supplementary Table 5—Example MedWAS Data: Example instances in outputted MedWAS data set

Phenotype	Drug	OR	*p*	n_total	n_cases	n_controls	Description	Group
008	ABACAVIR	0.931	0.631	45433	826	44607	Intestinal infection	Infectious diseases
038	ABACAVIR	0.000159	0.972	45951	211	45740	Septicemia	Infectious diseases
054	ABACAVIR	0.000138	0.984	35117	114	35003	Herpes simplex	Infectious diseases

6. To count the number of drug exposure and no drug exposure from phenotype and control, write script in your preferred programming language to scan the phenotype data and medication data files for each participating mother-baby pair and then merge medication counts underlying each associated drug with above MedWAS outcome to obtain a harmonized data set.

Supplementary Results

Please see the accompanying Supplementary Data 1 file.

Supplementary Discussion

Below (in Supplementary Table 6), we provide a summary description of the demographics of our cohort of mothers and infants. The format of Supplementary Table 6 aligns with that mandated by the United States Public Health Service (PHS) for reporting race, ethnicity, and biological sex[3].

Supplementary Table 6—Racial, Ethnic, and Sexual Diversity of Mothers and Babies: Counting mothers and babies per PHS-specified demographic criteria reveals the inherent diversity of our data set, given the obstetric population at a large, urban academic medical center. In the interest of data security, we have marked the counts for categories with at most 5 people as "<=5," to reduce patient re-identifiability risk.

**Racial Categories**	**Ethnic Categories**									
	Not Hispanic or Latino			Hispanic or Latino			Unknown/Not Reported Ethnicity			**Total**
	**Female**	**Male**	**Unknown/Not Reported**	**Female**	**Male**	**Unknown/Not Reported**	**Female**	**Male**	**Unknown/Not Reported**	
American Indian/Alaska Native	133	49	<=5	170	41	<=5	13	<=5	<=5	406
Asian	3,221	1,364	<=5	82	27	<=5	92	24	<=5	4,810
Native Hawaiian or Other Pacific Islander	<=5	<=5	<=5	<=5	<=5	<=5	<=5	<=5	<=5	<=5
Black or African American	11,839	5,015	<=5	154	92	<=5	235	97	<=5	17,433
White	37,390	15,279	<=5	5,564	2,269	<=5	961	345	<=5	61,816
More than One Race	1,532	676	<=5	1,129	476	<=5	24	9	<=5	3,846
Unknown or Not Reported	993	468	<=5	1,792	820	<=5	1,854	625	<=5	6,561
**Total**	55,108	22,851	7	8,891	3,725	<=5	3,179	1,100	8	94,872

Supplementary References

1. The value of SNOMED CT. *SNOMED* https://www.snomed.org/snomed-ct/why-snomed-ct.
2. OMOP Common Data Model – OHDSI. https://www.ohdsi.org/data-standardization/the-common-data-model/.
3. Instructions and Form Files for PHS 398. https://grants.nih.gov/grants/funding/phs398/phs398.html.
