# Supplementary material for: Medication history-wide association studies for pharmacovigilance of pregnant patients
Source: Commun Med (Lond). 2022 Sep 16;2:115. doi: 10.1038/s43856-022-00181-w (PMC9481638; doi:10.1038/s43856-022-00181-w)
Supplement: Supplementary file 2 — Supplementary Data 1 [file 43856_2022_181_MOESM2_ESM.pdf]

MEDGROUP	SUBJECT COUNT
5	21593
5-	500
6 MP	<=5
Abacavir	29
Abatacept	<=5
Ac	19092
Acarbose	<=5
Acebutolol	8
Acetamide	15
Acetaminophen	7297
Acetate	737
Acetazolamide	13
Acetic acid	<=5
Acetylcysteine	9
Acitretin	<=5
Acyclovir	1016
Adalimumab	<=5
Adapalene	7
Adenosine	16
age	1575
Air	540
Al	19378
Albendazole	<=5
Albuterol	1667
Alclometasone	<=5
Alcohol	7
Aldosterone	<=5
Alendronate	12
Alfuzosin	<=5
Allopurinol	23
Almond	20
Aloe	<=5
Alprazolam	104
Alprostadil	<=5
Alteplase	31
Aluminum chloride	<=5
Aluminum hydroxide	<=5
Amantadine	<=5
Amber	13
Amiloride	<=5
Amino acids	7
Aminocaproic Acid	6

Aminophylline	<=5
Amiodarone	20
Amitriptyline	141
Amlodipine	84
Ammonia	9597
Ammonium lactate	19
Amoxicillin	1671
AMP	1346
Amphetamine	39
Amphotericin B	<=5
Ampicillin	1098
Amprenavir	<=5
Anastrozole	<=5
Antipyrine	15
APAP	407
Apple	52
Aprepitant	<=5
Argatroban	<=5
Arginine	<=5
Aripiprazole	23
AS	10309
ASC	256
Ascorbic acid	106
Asenapine	<=5
Asp	1418
Aspart	279
Aspirin	1150
Astaxanthin	<=5
Atazanavir	53
Atenolol	97
Atomoxetine	<=5
Atorvastatin	46
Atovaquone	<=5
Atracurium	<=5
Atropine	45
ATT	52
AVAC	76
axin	119
Azathioprine	40
Azelaic acid	11
Azelastine	54
Azithromycin	3697
Aztreonam	8

Bacitracin	106
Baclofen	42
Balsalazide	<=5
Barbital	14
Barium	122
Barium sulfate	116
BCG vaccine	<=5
Bean	404
Beclomethasone dipropionate	<=5
Belimumab	<=5
Belladonna	11
Benazepril	22
Benzatropine	<=5
Benzocaine	3598
Benzodiazepine	10
Benzonatate	238
Benzoyl peroxide	47
Benzoylecgonine	<=5
Bepotastine	<=5
Beta carotene	<=5
Betamethasone	3370
Betaxolol	<=5
Bethanechol	<=5
Bevacizumab	<=5
Bexarotene	<=5
BIA	20
Bimatoprost	<=5
Biotin	61
Bisacodyl	137
Bismuth Subsalicylate	41
Bisoprolol	<=5
Bivalirudin	<=5
Black cohosh	<=5
Black walnut	<=5
BNP	<=5
Borage oil	<=5
Boric acid	<=5
Botulinum Toxin Type A	7
Botulinum Toxin Type B	<=5
Brimonidine	<=5
Bromfenac	<=5
Bromocriptine	9
Brompheniramine	<=5

Budesonide	84
Bumetanide	15
Bupivacaine	7221
Buprenorphine	321
Bupropion	164
Buspirone	78
Butalbital	547
Butamben	<=5
Butoconazole	<=5
Butorphanol	4494
Cabergoline	16
Cadmium	<=5
Caffeine	1645
Calciferol	340
Calcio	<=5
Calcitriol	7
Calcium	2707
Calcium acetate	9
Calcium Carbonate	1579
Calcium Chloride	11
Calcium Citrate	56
Calcium gluconate	37
Calcium polycarbophil	<=5
Camphor	9
Canagliflozin	<=5
Candida albicans	6
Capsaicin	<=5
Captopril	<=5
Carbamazepine	25
Carbamide	7
Carbamide peroxide	7
Carbazochrome	<=5
Carbidopa	<=5
Carbon dioxide	49
Carbon monoxide	<=5
Carbonate	1645
Carbonyl iron	<=5
Carboplatin	<=5
Carboprost tromethamine	325
Carisoprodol	20
Carrot	<=5
Carvedilol	46
Castor oil	9

Cauliflower	9
CD	36
Cefaclor	<=5
Cefazolin	2101
Cefdinir	141
Cefepime	23
Cefixime	19
Cefotaxime	<=5
Cefoxitin	29
Cefpodoxime	16
Cefprozil	6
Ceftaroline fosamil	<=5
Ceftazidime	6
Ceftizoxime	<=5
Ceftriaxone	664
Cefuroxime	37
Celecoxib	<=5
Cellulose	<=5
Cephalexin	876
Cephradine	22
Certolizumab pegol	<=5
Cetirizine	272
Cetylpyridinium	17
Cevimeline	<=5
Chamomile	<=5
Cherry	169
Chicken	<=5
Chloral hydrate	<=5
Chlordiazepoxide	<=5
Chlorhexidine	57
Chloride	4879
Chlorophyll	<=5
Chloroprocaine	41
Chloroquine	65
Chlorothiazide	231
Chloroxylenol	24
Chlorphenoxamine	<=5
Chlorpromazine	13
Chlorthalidone	13
Chlorzoxazone	<=5
Cholecalciferol	156
Cholestyramine	14
Cholic Acid	6

Choline	138
Chondroitin sulfate	<=5
Chromium picolinate	<=5
Cicl	33
Ciclopirox	21
Cilostazol	<=5
Cimetidine	39
Cinnamon	25
Ciprofloxacin	88
Cisatracurium	<=5
Cisplatin	<=5
Citalopram	272
Citric Acid	222
Clarithromycin	8
Clavulanate	395
Clidinium	<=5
Clindamycin	949
Clobazam	<=5
Clobetasol	90
Clobetasol propionate	<=5
Clomipramine	<=5
Clonazepam	85
Clonidine	762
Clopidogrel	19
Clorazepate	<=5
Clotrimazole	228
Clove	<=5
Cloxacillin	46
Clozapine	<=5
Cobalamin	108
Cocaine	97
Cocoa	9
Cocoa butter	9
Coconut	15
Coconut oil	7
Cod	2402
Cod liver oil	14
Codeine	282
Colace	2917
Colchicine	12
Colestipol	<=5
Colistimethate	<=5
Colistin	<=5

Collagenase clostridium histolyticum	<=5
Conjugated estrogens	<=5
COP	482
Copper	30
Corn	<=5
Corticotropin	<=5
Cortisone	6940
Cortisone acetate	87
Cranberry	37
Cucumber	10
Curcumin	<=5
Cyanocobalamin	103
Cycl	2007
Cyclobenzaprine	758
Cyclopentolate	<=5
Cyclophosphamide	6
Cyclosporine	10
Cyproheptadine	8
Cysteine	14
Cytarabine	<=5
d-amphetamine	<=5
Danazol	<=5
Dantrolene	<=5
Dapsone	9
Daptomycin	6
Darbepoetin	<=5
Darbepoetin alfa	<=5
Darunavir	35
Date	315
Daunorubicin	<=5
DCA	<=5
DCI	<=5
dDAVP	21
Deferasirox	<=5
Deferoxamine	<=5
Deoxycholic Acid	6
Desipramine	<=5
Desmopressin	18
Desogestrel	8
Desonide	126
Desoximetasone	6
Desvenlafaxine	<=5
Detemir	7

Dexamethasone	271
Dexlansoprazole	<=5
Dexmedetomidine	<=5
Dextran	14
Dextroamphetamine	19
Dextromethorphan	76
Dextrose	2331
DHA	595
Diatrizoate	<=5
Diazepam	143
Diclofenac	40
Dicloxacillin	46
Dicyclomine	54
Diflorasone	<=5
Diflucan	2290
Digoxin	47
Diltiazem	48
Dimenhydrinate	<=5
Dimethicone	<=5
Diphenhydramine	1924
Diphenoxylate	25
Dipyridamole	<=5
DM	96
Dobutamine	<=5
Docetaxel	<=5
Docusate	4157
Dofetilide	<=5
Dolasetron	143
Dolutegravir	12
Domperidone	<=5
Donepezil	<=5
Dopamine	<=5
Doripenem	<=5
Dornase alfa	<=5
Dorzolamide	<=5
Doxazosin	8
Doxepin	19
Doxorubicin	<=5
Doxycycline	174
Doxylamine	658
Dronabinol	<=5
Droperidol	34
Drospirenone	6

Duloxetine	40
Dutasteride	<=5
Dydrogesterone	<=5
Ecgonine	<=5
Echinacea	<=5
Econazole	15
Efavirenz	<=5
elate	<=5
Elm	12
Emtricitabine	70
Enalapril	22
Enalaprilat	<=5
Enoxaparin	307
Entacapone	<=5
Ephedrine	526
Epinastine	<=5
Epinephrine	2034
Eplerenone	<=5
Epoprostenol	<=5
Ergocalciferol	203
Ertapenem	17
Erythromycin	2410
Erythropoietin	<=5
Escitalopram	83
Esmolol	12
Esomeprazole	106
Estradiol	158
Estradiol valerate	<=5
Estriol	23
Eszopiclone	<=5
Etanercept	<=5
Ethambutol	<=5
Ethanol	11
Ethosuximide	<=5
Ethyl chloride	<=5
Ethynodiol diacetate	<=5
Etiracetam	91
Etodolac	19
Etomidate	11
Etonogestrel	<=5
Etravirine	<=5
Evening primrose oil	24
Everolimus	<=5

Exemestane	<=5
Ezetimibe	<=5
Factor VIII	12
Famciclovir	<=5
Famotidine	3589
Felbamate	<=5
Felodipine	<=5
Fenofibrate	11
Fentanyl	7281
Ferric Carboxymaltose	<=5
Ferric subsulfate	7
Ferrous asparto glycinate	7
Ferrous bisglycinate	<=5
Ferrous fumarate	655
Ferrous gluconate	81
Ferumoxytol	<=5
Fexofenadine	53
Finasteride	13
Fish oil	407
Flavoxate	<=5
Flax seed	6
Flecainide	31
Fluconazole	2101
Fludrocortisone	15
Flumazenil	<=5
Flunisolide	6
Fluocinolone	10
Fluocinolone acetonide	<=5
Fluocinonide	44
Fluorescein	26
Fluorometholone	<=5
Fluorouracil	<=5
Fluoxetine	156
Fluphenazine	<=5
Flur	32
Fluticasone	680
Fluticasone furoate	10
Fluticasone propionate	15
Fluvastatin	<=5
Fluvoxamine	<=5
Folate	203
Folic Acid	3233
Fondaparinux	<=5

Formoterol	<=5
Fosamprenavir	<=5
Fosaprepitant	<=5
Fosfomycin	9
Fosinopril	<=5
Fosphenytoin	<=5
Fructose	<=5
FTP	23
Furosemide	171
Gabapentin	267
Gadobutrol	<=5
Gadolinium	<=5
Gadoxetate	<=5
Galantamine	<=5
Ganciclovir	7
Garlic	<=5
Gelatin	<=5
Gemfibrozil	8
Gentamicin	587
Ginger	91
Ginkgo biloba	<=5
Ginseng	<=5
Glimepiride	18
Glipizide	65
Glu	1002
Glucagon	433
Glucosamine	26
Glucose	215
Glutathione	<=5
Glyburide	1080
Glycerin	167
Glycine	<=5
Glycolic acid	<=5
GnRH	<=5
Gold	<=5
Granisetron	<=5
Grape	18
Grapefruit	<=5
Guaifenesin	319
Guanfacine	11
Haloperidol	190
Halothane	<=5
Heparin	2086

Hepatitis A Vaccine	8
Hepatitis B Vaccine (Recombinant)	9
Hetastarch	21
Histamine	30
Homatropine	<=5
Honey	<=5
Human insulin	783
Hyaluronate	<=5
Hyaluronidase	<=5
Hydralazine	271
Hydrochlorothiazide	231
Hydrocodone	1003
Hydrocortisone	6910
Hydrocortisone acetate	87
Hydrocortisone butyrate	<=5
Hydrocortisone valerate	18
Hydrogen peroxide	<=5
Hydromorphone	453
Hydroquinone	7
Hydroxide	2254
Hydroxocobalamin	<=5
Hydroxychloroquine	64
Hydroxyprogesterone	206
Hydroxyprogesterone caproate	155
Hydroxyurea	9
Hydroxyzine	1056
Hyoscyamine	182
Hypromellose	6
Ib	1905
Ibuprofen	1697
Idarubicin	<=5
IMD	26
Imipramine	<=5
Imiquimod	14
Indapamide	10
Indinavir	<=5
Indole	<=5
Infliximab	11
Influenza A virus	<=5
INH	9522
Inosine	<=5
Inositol	<=5
Insulin Aspart	197

Insulin Detemir	7
Insulin Glargine	306
Insulin Human	818
Insulin Lispro	212
Inulin	<=5
Iodide	<=5
Iodine	45
Iodoform	<=5
Iohexol	26
iol	314
ione	2020
Ipratropium	228
Irbesartan	<=5
Iron	2713
Iron Dextran	13
Isometheptene	6
Isoniazid	14
Isosorbide	34
Isosorbide dinitrate	<=5
Isosorbide Mononitrate	26
Isotretinoin	<=5
Isoxsuprine	<=5
Itraconazole	<=5
Ivermectin	<=5
IVIg	25
Kale	56
KCl	494
Ketamine	60
Ketoconazole	74
Ketoprofen	6
Ketorolac	219
Kidney bean	<=5
Labetalol	1317
Lacosamide	<=5
Lactic Acid	<=5
Lactobacillus reuteri	<=5
Lactose	13
Lactulose	52
Lamivudine	65
Lamotrigine	114
Lanolin	40
Lansoprazole	97
Latanoprost	9

Ledipasvir	<=5
Leflunomide	<=5
Lemon	39
Letrozole	41
Leucovorin	<=5
Leuprolide	<=5
Levetiracetam	91
Levobunolol	<=5
Levocarnitine	<=5
Levocetirizine	13
Levodopa	17
Levofloxacin	40
Levonorgestrel	96
Levothyroxine	844
Lidocaine	10739
Linaclotide	<=5
Lindane	<=5
Linezolid	<=5
Liothyronine	8
Lipoic Acid	<=5
Lisdexamfetamine	<=5
Lisinopril	296
Lithium carbonate	22
Lithium citrate	<=5
Loperamide	289
Lopinavir	22
Loratadine	508
Lorazepam	225
Losartan	41
Lovastatin	16
Lubiprostone	<=5
Lurasidone	<=5
Lutein	<=5
Mafenide	<=5
Magnesia	1439
Magnesium	3888
Magnesium chloride	<=5
Magnesium citrate	61
Magnesium gluconate	8
Magnesium hydroxide	1595
Magnesium oxide	167
Magnesium sulfate	2515
Malic Acid	<=5

Maltose	6
Manganese	<=5
Mango	7
Mannitol	<=5
Maprotiline	<=5
MCC	<=5
Mebendazole	<=5
Meclizine	157
Medroxyprogesterone acetate	10
Mefenamic acid	<=5
Mefloquine	7
Melatonin	290
Meloxicam	48
Memantine	<=5
Meperidine	309
Mepivacaine	7
Meprobamate	<=5
Mercaptopurine	9
Meropenem	11
Mesalamine	17
Metaraminol	<=5
Metaxalone	<=5
Metformin	542
Methadone	180
Methenamine	<=5
Methimazole	40
Methocarbamol	10
Methotrexate	57
Methyclothiazide	<=5
Methyl salicylate	7
Methylcellulose	<=5
Methylcobalamin	<=5
Methyldopa	208
Methylnaltrexone	<=5
Methylosterone	<=5
Methylphenidate	12
Methylprednisolone	194
Metoclopramide	958
Metolazone	8
Metoprolol	318
Metronidazole	2093
Metyrapone	<=5
Mexiletine	<=5

Micafungin	<=5
Miconazole	191
Midazolam	207
Midodrine	14
Mifepristone	<=5
Milk of magnesia	1436
Milk thistle	<=5
Milrinone	<=5
Mineral oil	52
Minocycline	17
Minoxidil	<=5
Mirtazapine	61
Misoprostol	5172
MoCo	412
Modafinil	<=5
Molybdenum	<=5
Mometasone	32
Mometasone furoate	<=5
Montelukast	98
Morphine	2680
Moxifloxacin	7
MPA	658
Mupirocin	102
Mycophenolate	13
Mycophenolate mofetil	12
Mycophenolic acid	<=5
Nabumetone	6
NAC	381
Nadolol	10
Nafcillin	39
Nalbuphine	2667
Naloxone	1490
Naltrexone	<=5
Naproxen	96
Naratriptan	<=5
Natalizumab	<=5
Nebivolol	<=5
Nefazodone	<=5
Nelfinavir	8
Neomycin	46
Neon	1989
Neostigmine	54
Nevirapine	<=5

Niacin	18
Nicardipine	15
Nicotine	511
Nifedipine	629
Nitazoxanide	<=5
Nitrate	162
Nitric Oxide	<=5
Nitrofurantoin	2226
Nitrogen	78
Nitroglycerin	106
Nitrous oxide	148
Nizatidine	<=5
Non	189
Nor	2497
Norelgestromin	<=5
Norepinephrine	12
Norgestimate	8
Norgestrel	107
Nortriptyline	21
Nystatin	375
Octreotide	<=5
Ofloxacin	135
Okra	<=5
Olanzapine	102
Olive oil	<=5
Olopatadine	<=5
Omalizumab	<=5
Omega-3	54
Omega-3 fatty acids	6
Omeprazole	940
OMO	505
omycin	6003
Ondansetron	9459
Opium	241
Orange	145
Oregano	<=5
Orlistat	<=5
Orphenadrine	<=5
Oseltamivir	197
Oss	18
osterone	<=5
osterone cypionate	<=5
osterone enanthate	<=5

Oxacillin	47
Oxazepam	<=5
Oxcarbazepine	126
Oxybutynin	76
Oxycodone	1127
Oxygen	30
Oxymetazoline	29
Oxymorphone	15
Oxytocin	11736
Oyster	13
Ozone	<=5
Paclitaxel	<=5
Pancrelipase	<=5
Pancuronium	<=5
Pantoprazole	266
Pantothenic acid	<=5
Papaverine	<=5
Papaya	<=5
Paprika	<=5
Paroxetine	28
PAS	285
PAT	269
pC	2428
PCA	90
Pea	25
Peanut	<=5
Peanut oil	<=5
Pearl	<=5
Pentamidine	<=5
Pentazocine	<=5
Pentoxifylline	<=5
Peppermint oil	<=5
Pepsin	<=5
Perazine	728
Perflutren	<=5
Permethrin	81
Perphenazine	<=5
Pertuzumab	<=5
Petrolatum	<=5
Phenazopyridine	215
Phencyclidine	<=5
Pheniramine	22
Phenobarbital	14

Phenol	21
Phentermine	18
Phenylephrine	1357
Phenytoin	17
Phloroglucin	<=5
Phloroglucinol	<=5
Phosphate	70
Phosphatidyl serine	<=5
Pilocarpine	<=5
Pindolol	<=5
Pineapple	<=5
Pioglitazone	<=5
Piperacillin	67
Piperazine	<=5
Pirbuterol	<=5
Pitavastatin	<=5
Platinum	<=5
Podofilox	<=5
Polycarbophil	<=5
Polyethylene glycol	342
Polymyxin B Sulfate	12
Polystyrene sulfonate	<=5
Polyvinyl alcohol	<=5
Pomegranate	<=5
Poractant alfa	<=5
Pork	224
Potassium	4555
Potassium acetate	8
Potassium bicarbonate	<=5
Potassium Chloride	710
Potassium Citrate	6
Potassium gluconate	9
Potassium Iodide	<=5
Povidone	<=5
PPD	782
Pramipexole	<=5
Pramoxine	6270
Pravastatin	48
Praziquantel	<=5
Prazosin	35
Prednisolone	220
Prednisone	561
Prednisone acetate	<=5

Pregabalin	8
Prilocaine	46
Primidone	6
Primrose oil	32
Probenecid	<=5
Procainamide	<=5
Procaine	43
Prochlorperazine	727
Progesterone	1146
Proguanil	<=5
Promazine	13
Promethazine	5654
Propafenone	<=5
Proparacaine	32
Propofol	281
Propranolol	82
Propylene glycol	<=5
Propylthiouracil	35
Protamine	8
Protein C	<=5
Prothrombin	45
Pseudoephedrine	71
Psyllium	32
PV	19
Pyrantel	<=5
Pyrazinamide	<=5
Pyrethrins	<=5
Pyridostigmine	8
Pyridoxal	<=5
Pyridoxine	561
Pyrimethamine	<=5
Quetiapine	78
Quinapril	<=5
Quinine	<=5
quinol	12
Rabeprazole	<=5
Rabies immune globulin	<=5
Racepinephrine	6
Raltegravir	12
Ramipril	24
Ranibizumab	<=5
Ranitidine	725
Raspberry	<=5

Regadenoson	<=5
Remifentanil	610
Resveratrol	<=5
Retinol	<=5
Ribavirin	<=5
Riboflavin	23
Rice	461
Rifabutin	<=5
Rifapentine	<=5
Rilpivirine	<=5
rina	21
Risperidone	44
Ritonavir	97
Rituximab	7
Rivastigmine	<=5
Rizatriptan	8
Rocephin	589
Rocuronium	58
Ropinirole	7
Ropivacaine	553
Rosin	6
Rosuvastatin	<=5
Rotavirus Vaccine	<=5
Royal jelly	<=5
Rutin	<=5
Sage	<=5
Salbutamol	<=5
Salicylic acid	8
Salmeterol	75
Salmon	<=5
Salsalate	<=5
Saquinavir	<=5
Scopolamine	54
Selegiline	<=5
Selenium	276
Selenium Sulfide	<=5
Senna	159
Sennosides	25
Serine	<=5
Sertraline	483
Sesame oil	<=5
Sesame seed	<=5
Sevelamer	<=5

Sildenafil	7
Silver	60
Silver nitrate	6
Silver sulfadiazine	31
Simethicone	2280
Simvastatin	71
Sirolimus	<=5
Sitagliptin	<=5
SMX	42
Sodium acetate	<=5
Sodium bicarbonate	40
Sodium Chloride	4213
Sodium Citrate	221
Sodium fluoride	<=5
Sodium lauryl sulfate	<=5
Sodium oxybate	<=5
Sofosbuvir	<=5
Sotalol	35
Spiramycin	<=5
Spironolactone	52
Squash	7
Sterile water	31
Succinate	364
Succinic acid	<=5
Succinylcholine	131
Sucralfate	44
Sucrose	28
Sufentanil	<=5
Sugammadex	12
Sulbactam	20
Sulfacetamide	15
Sulfadiazine	31
Sulfamethoxazole	211
Sulfasalazine	<=5
Sulfate	6917
Sulindac	<=5
Sumatriptan	90
Synephrine	<=5
T3	7
Tacrolimus	63
Talc	53
Talopram	272
Tamoxifen	<=5

Tamsulosin	81
Tapentadol	<=5
Tazobactam	68
Tea tree oil	<=5
Telbivudine	<=5
Telmisartan	<=5
Temazepam	10
Tenofovir	80
Tenofovir alafenamide	<=5
Tenofovir disoproxil	20
Terazosin	11
Terbinafine	15
Terbutaline	7738
Terconazole	864
Tetanus toxoid	9
Tetracaine	28
Tetracycline	6
Tetrofosmin	<=5
Theanine	<=5
Theophylline	8
Thiamine	349
Thiopental	<=5
Thrombin	62
Thyroglobulin	<=5
Timolol	11
Tinidazole	<=5
Tioconazole	<=5
Tiotropium	<=5
Titanium	<=5
Tizanidine	30
TMP	43
Tobramycin	29
Tolnaftate	<=5
Tolterodine	<=5
Topiramate	57
tPA	32
Tramadol	86
Trandolapril	<=5
Tranexamic acid	25
Tranylcypromine	<=5
Trazodone	157
Tretinoin	62
Triamcinolone	489

Triamterene	14
Triazolam	<=5
Trihexyphenidyl	<=5
Trimethobenzamide	<=5
Trimethoprim	276
Tromethamine	468
Tropicamide	<=5
Trospium	<=5
Trypsin	<=5
Turmeric	<=5
Tyramine	14
Ubiquinol	6
UDCA	<=5
Uracil	38
Urea	20
Urethane	<=5
Uric Acid	208
Ursodeoxycholic acid	<=5
Ursodiol	128
Ustekinumab	<=5
Val	2391
Valerian	<=5
Valganciclovir	<=5
Valproic Acid	<=5
Valsartan	<=5
Vanadium	<=5
Vancomycin	285
Vanilla	<=5
Vardenafil	<=5
Vasopressin	<=5
Vecuronium	79
Vedolizumab	<=5
Venlafaxine	63
Verapamil	37
Vilanterol	<=5
Vincristine	<=5
Vinegar	6
Vinorelbine	<=5
Vitamin A	23
Vitamin B1	324
Vitamin B12	67
Vitamin B2	13
Vitamin B6	511

Vitamin C	430
Vitamin D	993
Vitamin D2	236
Vitamin D3	392
Vitamin E	119
Von willebr	<=5
Voriconazole	<=5
Warfarin	117
Water	700
Wheat bran	<=5
Witch hazel	8
Zaleplon	<=5
Zeaxanthin	<=5
Zidovudine	134
Zinc	171
Zinc acetate	11
Zinc oxide	11
Zinc sulfate	<=5
Ziprasidone	7
Zn	<=5
Zolpidem	2123
Zonisamide	20
Zopiclone	<=5
