# Supplementary material for: Medication history-wide association studies for pharmacovigilance of pregnant patients
Source: Commun Med (Lond). 2022 Sep 16;2:115. doi: 10.1038/s43856-022-00181-w (PMC9481638; doi:10.1038/s43856-022-00181-w)
Supplement: Supplementary file 3 — Description of Additional Supplementary Files [file 43856_2022_181_MOESM3_ESM.pdf]

Description of Additional Supplementary Files

File name: Supplementary Data 1
Description: All pharmaceutical and nutraceutical agents documented in pregnant patients' EHRs, per the medication identification approaches we describe in our manuscript, alongside the number of EHRs within which each agent featured. From this raw, holistic list, we removed any strings apparent as components of larger drug names—the capture of which we considered artifact from the performance of our NLP tools. In the interest of patient data security, we have marked the counts for agents with at most 5 exposures as "<=5," to reduce patient reidentifiability risk.
