# Supplementary material for: Medication history-wide association studies for pharmacovigilance of pregnant patients
Source: Commun Med (Lond). 2022 Sep 16;2:115. doi: 10.1038/s43856-022-00181-w (PMC9481638; doi:10.1038/s43856-022-00181-w)
Supplement: Supplementary file 4 — Reporting Summary [file 43856_2022_181_MOESM4_ESM.pdf]

nature research

Corresponding author(s): David M. Aronoff (and Anup P. Challa)

Last updated by author(s): Aug. 26, 2022

Reporting Summary

Nature Research wishes to improve the reproducibility of the work that we publish. This form provides structure for consistency and transparency in reporting. For further information on Nature Research policies, see our Editorial Policies and the Editorial Policy Checklist.

Please do not complete any field with "not applicable" or n/a. Refer to the help text for what text to use if an item is not relevant to your study.
For final submission: please carefully check your responses for accuracy; you will not be able to make changes later.

Statistics

For all statistical analyses, confirm that the following items are present in the figure legend, table legend, main text, or Methods section.

n/a	Confirmed	
☐	☒	The exact sample size (*n*) for each experimental group/condition, given as a discrete number and unit of measurement
☐	☒	A statement on whether measurements were taken from distinct samples or whether the same sample was measured repeatedly
☐	☒	The statistical test(s) used AND whether they are one- or two-sided<br>*Only common tests should be described solely by name; describe more complex techniques in the Methods section.*
☐	☒	A description of all covariates tested
☐	☒	A description of any assumptions or corrections, such as tests of normality and adjustment for multiple comparisons
☒	☐	A full description of the statistical parameters including central tendency (e.g. means) or other basic estimates (e.g. regression coefficient) AND variation (e.g. standard deviation) or associated estimates of uncertainty (e.g. confidence intervals)
☐	☒	For null hypothesis testing, the test statistic (e.g. *F*, *t*, *r*) with confidence intervals, effect sizes, degrees of freedom and *P* value noted<br>*Give P values as exact values whenever suitable.*
☒	☐	For Bayesian analysis, information on the choice of priors and Markov chain Monte Carlo settings
☒	☐	For hierarchical and complex designs, identification of the appropriate level for tests and full reporting of outcomes
☐	☒	Estimates of effect sizes (e.g. Cohen's *d*, Pearson's *r*), indicating how they were calculated

Our web collection on statistics for biologists contains articles on many of the points above.

Software and code

Policy information about availability of computer code

Data collection	We leveraged a suite of previously published natural language processing (NLP) tools to extract phenotypic attributes and maternal drug exposures from narrative EHR data among all patients within the EHR databank at Vanderbilt University Medical Center (VUMC) who met our inclusion criteria. These tools included a general-purpose NLP tool (the 2015-indexed version of KnowledgeMap concept identifier (KMCI), available through https://www.vumc.org/cpm/cpm-blog/kmci-knowledgemap-concept-indexer), ML-based clinical note section tagger (the 2010-indexed version of SecTag, available to download at https://www.vumc.org/cpm/cpm-blog/sectag-tagging-clinical-note-section-headers), and version 1.3 of MedEx, an NLP algorithm for identifying medication exposures within free clinical text (available to download at https://sbmi.uth.edu/ccb/resources/medex.htm). Source papers describing the mechanisms of these tools are cited in the manuscript text. Data manually sourced from clinical decision support systems were available through VUMC's institutional subscriptions to these fora.
Data analysis	The PheWAS package in R (version 0.12.3) is open-source and available through GitHub (https://github.com/PheWAS/PheWAS). We used version 3.6.3 of R for executing this package. Source papers describing PheWAS mechanisms are cited in the manuscript text.

For manuscripts utilizing custom algorithms or software that are central to the research but not yet described in published literature, software must be made available to editors and reviewers. We strongly encourage code deposition in a community repository (e.g. GitHub). See the Nature Research guidelines for submitting code & software for further information.

Data

Policy information about availability of data

All manuscripts must include a data availability statement. This statement should provide the following information, where applicable:
- Accession codes, unique identifiers, or web links for publicly available datasets
- A list of figures that have associated raw data
- A description of any restrictions on data availability

Disclosure of our MedWAS data, though de-identified and aggregated, is subject to approval and oversight by the Office of Contracts Management (OCM) at VUMC, as our source data are derived from protected health information (PHI), and some drug-disease pairs are individually re-identifiable. Therefore, institutional policies prevented us from publicly releasing our data tables and their annotations in the interest of patient security, but, within the data sharing regulations of our

institution, we attempted to provide meaningful information on the content and formatting of our outputs throughout this manuscript. We are committed to open-source science and to ensuring the reproducibility of the research we present here; therefore, we are happy to discuss data transfer requests with researchers interested in our results. Interested investigators should contact the Corresponding Authors at the addresses accompanying our manuscript, and they are happy to discuss forwarding such requests to OCM (towards a data use agreement) within 30 days of receiving such a collaboration request.

Field-specific reporting

Please select the one below that is the best fit for your research. If you are not sure, read the appropriate sections before making your selection.

☒ Life sciences ☐ Behavioural & social sciences ☐ Ecological, evolutionary & environmental sciences

For a reference copy of the document with all sections, see nature.com/documents/nr-reporting-summary-flat.pdf

Life sciences study design

All studies must disclose on these points even when the disclosure is negative.

Sample size	We accessed all EHRs at VUMC matching the inclusion criteria we describe in our manuscript, providing us with the maximum possible sample size. This is a customary approach to facilitate retrospective analysis of medical records.
Data exclusions	When curating our cohorts, we did not exclude EHRs otherwise matching our inclusion criteria.
Replication	The high quality of the EHR databank at VUMC, the NLP tools we used for data extraction, and the outcomes reported by the PheWAS procedure are all well-established in previous literature. Results from our MedWAS model were validated against existing clinical literature on drug safety in pregnancy and a subsequent consensus review procedure among expert clinicians and disciplinary scientists; proof-of-concept in a pilot study informed progression to full-stage research. Source EHR data were available to confirm accurate ascertainment of relevant EHR data fields by our NLP tools.
Randomization	As described in the manuscript, we considered that real-world obstetric practice assigns patients with the same disease to treatment vs. no treatment; we corrected post hoc for potentially associated covariates and confounders, to prioritize our model's results.
Blinding	This was a retrospective study with no intervention, so blinding was not relevant to our study design.

Reporting for specific materials, systems and methods

We require information from authors about some types of materials, experimental systems and methods used in many studies. Here, indicate whether each material, system or method listed is relevant to your study. If you are not sure if a list item applies to your research, read the appropriate section before selecting a response.

Materials & experimental systems

n/a	Involved in the study	
☒	☐	Antibodies
☒	☐	Eukaryotic cell lines
☒	☐	Palaeontology and archaeology
☒	☐	Animals and other organisms
☒	☐	Human research participants
☐	☒	Clinical data
☒	☐	Dual use research of concern

Methods

n/a	Involved in the study	
☒	☐	ChIP-seq
☒	☐	Flow cytometry
☒	☐	MRI-based neuroimaging

Clinical data

Policy information about clinical studies

All manuscripts should comply with the ICMJE guidelines for publication of clinical research and a completed CONSORT checklist must be included with all submissions.

Clinical trial registration	This was not a prospective clinical study and therefore did not require national registration.
Study protocol	This was not a prospective clinical study. All relevant protocol elements are disclosed within our manuscript and its accompanying supplementary information.
Data collection	All EHRs collected from healthcare encounters at VUMC until January 22, 2020 (date of last data pull before analysis)
Outcomes	ICD-9 and ICD-10 billing codes from the EHR, subsequently mapped to Phecodes

This checklist template is licensed under a Creative Commons Attribution 4.0 International License, which permits use, sharing, adaptation, distribution and reproduction in any medium or format, as long as you give appropriate credit to the original author(s) and the source, provide a link to the Creative Commons license, and indicate if changes were made. The images or other third party material in this article are included in the article's Creative Commons license, unless indicated otherwise in a credit line to the material. If material is not included in the article's Creative Commons license and your intended use is not permitted by statutory regulation or exceeds the permitted use, you will need to obtain permission directly from the copyright holder. To view a copy of this license, visit http://creativecommons.org/licenses/by/4.0/
